# Etiology of 305 cases of refractory hematospermia and therapeutic options by emerging endoscopic technology

**DOI:** 10.1038/s41598-019-41123-2

**Published:** 2019-03-22

**Authors:** Liang-Gong Liao, Yan-Feng Li, Yong Zhang, Ke Li, Tong Zhu, Bo-Jun Li, Qi Wang, Xu-Dong Liu, Yong Luo, Bo Zhou, Jun Jiang

**Affiliations:** 1 Department of Urology, Daping Hospital, Army Medical University, Chongqing, China; 2 Department of Gastrointestinal Surgery, Hubei Cancer Hospital, Tongji Medical College, University of Science and Technology, Huazhong, China; 3 Department of Urology, Suining Central Hospital, Suining, Sichuan China

## Abstract

To investigate the surgical outcomes of vesiculoscopy on refractory hematospermia and ejaculatory duct obstruction (EDO), the clinical data (including pelvic magnetic resonance imaging (MRI) examinations and the long-term effects of endoscopic treatment) from 305 patients were analyzed. Four main etiologic groups were found on MRI. We found that 62.0% (189/305) of patients showed abnormal signal intensity in MRI investigations in the seminal vesicle (SV) area. Cystic lesions were observed in 36.7% (112/305) of the patients. The third sign was dilatation or enlargement of unilateral or bilateral SV, which were observed in 32.1% (98/305) of the patients. The fourth sign was stone formation in SV or in an adjacent cyst, which was present in 8.5% (26/305) of the patients. The transurethral endoscopy or seminal vesiculoscopy and the related procedures, including fenestration in prostatic utricle (PU), irrigation, lithotripsy, stone removal, biopsy, electroexcision, fulguration, or transurethral resection/incision of the ejaculatory duct (TURED/TUIED), chosen according to the different situations of individual patients were successfully performed in 296 patients. Fenestrations in PU+ seminal vesiculoscopy were performed in 66.6% (197/296) of cases. Seminal vesiculoscopy via the pathological opening in PU was performed in 10.8% (32/296) of cases. TURED/TUIED + seminal vesiculoscopy was performed in 12.8% (38/296) of cases, and seminal vesiculoscopy by the natural orifices of the ejaculatory duct (ED) was performed in 2.4% (7/296) of cases. Electroexcision and fulguration to the abnormal blood vessels or cavernous hemangioma at posterior urethra were performed in 7.4% (22/296) of cases. Two hundred and seventy-one patients were followed up for 6–72 months. The hematospermia of all the patients disappeared within 2–6 weeks, and 93.0% of the patients showed no further hematospermia during follow-up. No obvious postoperative complications were observed. The transurethral seminal vesiculoscopy technique and related procedures are safe and effective approaches for refractory hematospermia and EDO.

## Introduction

Hematospermia, which is traditionally defined as the appearance of blood in the seminal fluid, has been recognized for centuries^1^. Hematospermia is a relatively uncommon symptom in urology and andrology, but its exact incidence is still unknown. Han reported that the incidence is 0.5% in a population attending screenings for prostate cancer^2^. Polito reported that hematospermia represented 1% of all symptoms in urinary system diseases^3^. The etiology of hematospermia can be classed into 10 categories based on the pathophysiological mechanisms of the hematospermia: (I) inflammatory, (II) infectious, (III) lithiasis, (IV) cystic, (V) obstructive, (VI) tumoral, (VII) vascular, (VIII) traumatic, (IX) iatrogenic, and (X) systemic origin^4^. Although it is a benign and self-limiting symptom in most patients, some patients who suffer from persistent or refractory hematospermia may indicate more serious underlying organic pathological changes, and may need further evaluation to relieve their considerable anxiety, stress, and concern. In the past few years, the development of seminal vesiculoscopy and related procedures has dramatically increased the accuracy of the diagnosis and the efficiency of the treatment of hematospermia^5–7^. However, this novel technology is not yet fully mature and has not become widely used, and the long-term curative effect has yet to be evaluated in a large-scale study. In fact, there are still many controversial questions or focused problems to be discussed, such as the method for identifying the opening of the ejaculatory duct (ED), the insertion approach for the seminal vesiculoscope, the characteristic magnetic resonance imaging (MRI) changes of refractory hematospermia and their management strategy, and the prevention of postoperative complications. With respect to these questions, the present study retrospectively summarized the clinical data of 305 patients with persistent or recurrent hematospermia or ejaculatory duct obstruction (EDO), their management techniques, and strategies with the emerging transurethral endoscopy. Our study adds unique information on these issues.

## Results

The patient age ranged from 20 to 77 years (mean age, 43.8 ± 11.5 years). The symptoms of hematospermia and infertility among these patients lasted from 6 to 120 months (mean, 39.6 ± 34.9 months). There were 134 patients <40 years of age and 171 patients ≥40 years of age. The principal symptoms were persistent or refractory hematospermia upon ejaculation, semen samples presented with bright or deep red color or as a coffee-like liquid, occasionally accompanied by blood clots. Most cases were not accompanied by any other symptoms, but a few patients experienced discomfort or pain in the lumbosacral or perineal region, ejaculatory pain, dysuria, urinary frequency and urgency, hematuria after sexual stimulation, loss of orgasmic intensity, azoospermia, oligoasthenozoospermia (Table 1).

**Table 1:** Symptoms of 305 patients with refractory hematospermia and/or EDO.

Symptoms	No. of patients	Percentage
Hematospermia	305	100.0
Urinary frequency or urgency	38	12.5
Dysuria	10	3.3
Hematuria after sexual stimulation	22	7.2
Pain or discomfort	21	6.9
Lumbosacral, perineal, or abdominal pain	12	3.9
Ejaculatory pain	9	3.0
Loss of orgasmic intensity	13	4.3
Azoospermia or oligoasthenozoospermia	19	6.2

**Figure 1:**
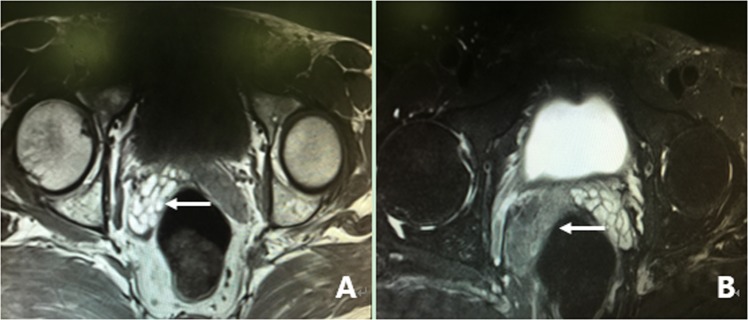
MRI image of a fresh hemorrhage in the right side of the SV. (**A**) This image shows high signal intensity in the right SV and low signal intensity in the left SV on T1WI. (**B**) This image shows low signal intensity in the right SV and high signal intensity in the left SV on T2WI. This pattern suggests that a fresh hemorrhage is present in the right SV.

**Figure 2:**
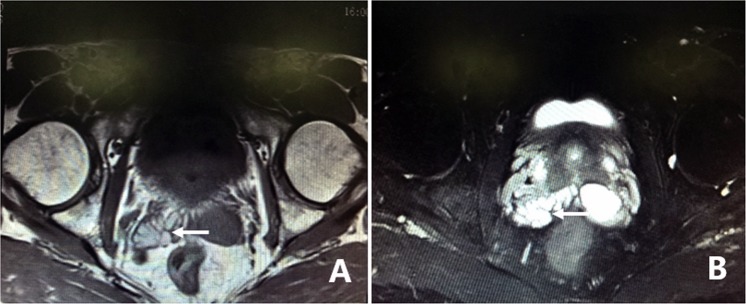
MRI image of an old hemorrhage in the right side of the SV. (**A**) This image shows high signal intensity in the right SV and low signal intensity in the left SV on T1WI. (**B**) This image shows similar high signal intensity in both sides of the SV on T2WI. This pattern suggests that an old hemorrhage is present in the right SV. There are slight dilatations and cystic changes in the right and left sides of the SV, respectively.

**Figure 3:**
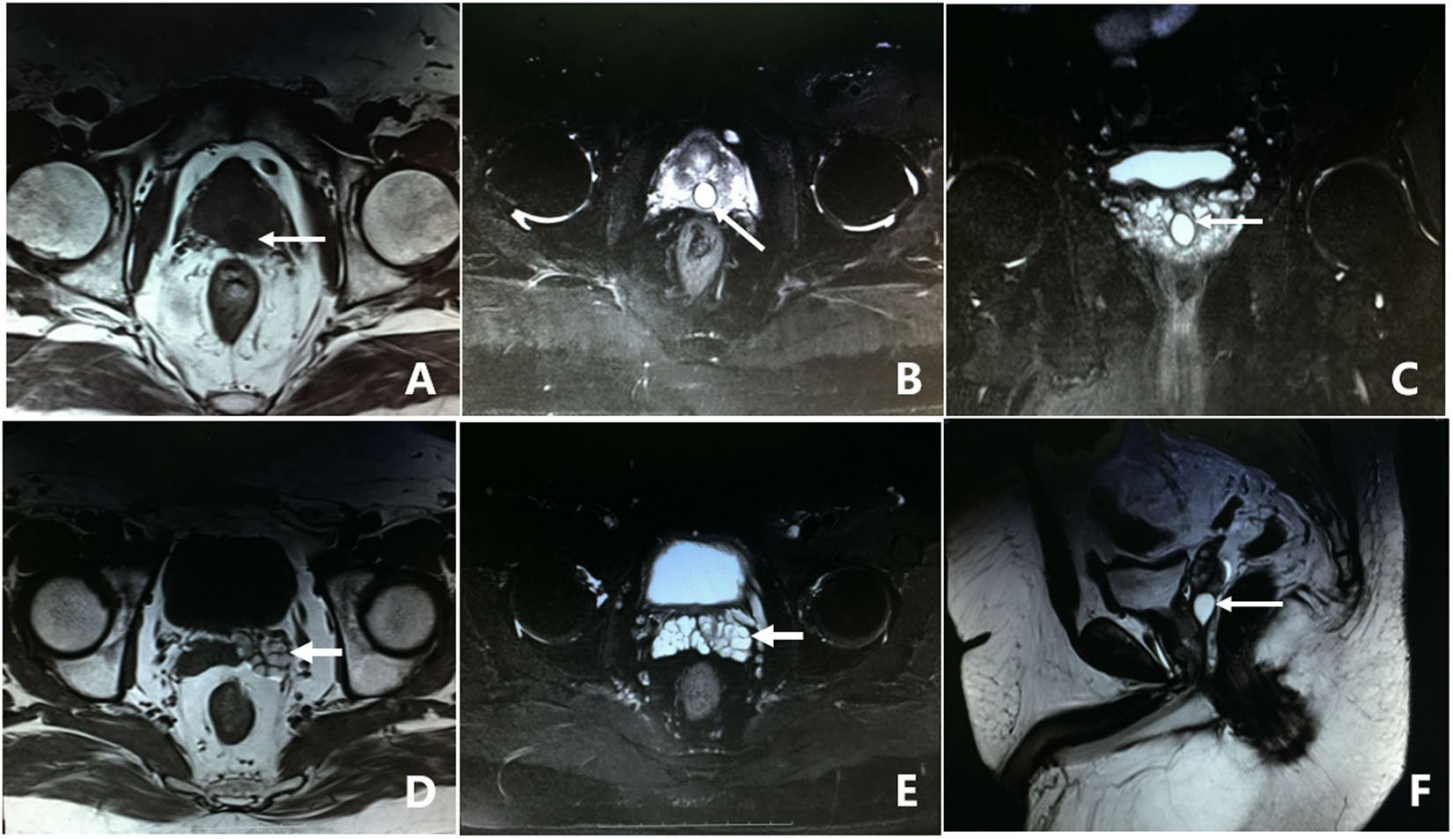
MRI image of PUC. (**A**) There is a 1.0 × 1.2 cm low-intensity signal cyst in the midline of the prostate on T1WI. (**B**,**C**,**F**) The cyst presents as high-intensity signal on T2WI. (**D**,**E**) The left SV presents as high signal intensity on both T1WI and T2WI, and its width has expanded to 2.1 cm. This pattern suggests that the cyst is a PUC and is accompanied by enlargement and an old hemorrhage in the left SV.

**Figure 4:**
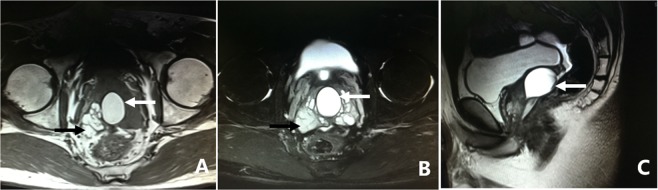
MRI image of MDC. (**A**) There is a 3.4 × 4.5 cm high-intensity signal cyst in the midline of the prostate, accompanied by dilation and high-intensity signals in the right SV on T1WI. (**B**) Both the cyst and the bilateral SV present as high-intensity signal on T2WI. (**C**) The cyst extended beyond the posterosuperior margin of the prostate gland on the sagittal plane. This pattern suggests that the cyst is MDC, accompanied by dilation and an old hemorrhage in the right SV.

**Figure 5:**
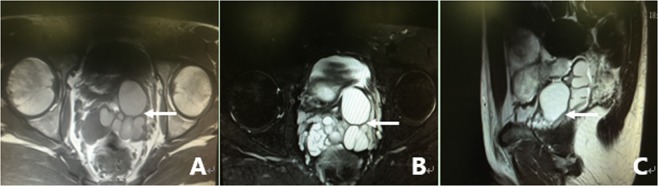
MRI image of SVC. (**A**) This image shows dilatation or enlargement in the left SV and presentation as a cystic structure with multiple cavities, with middle to high signal intensity in the left SV on T1WI. (**B**) This cyst presented as high signal intensity on T2WI, which is similar to that of the right SV. (**C**) The left SV presented as an obvious dilated cystic structure on the sagittal plane. This pattern suggests that the patient had a left SVC. There is also obvious dilation in the right SV.

**Figure 6:**
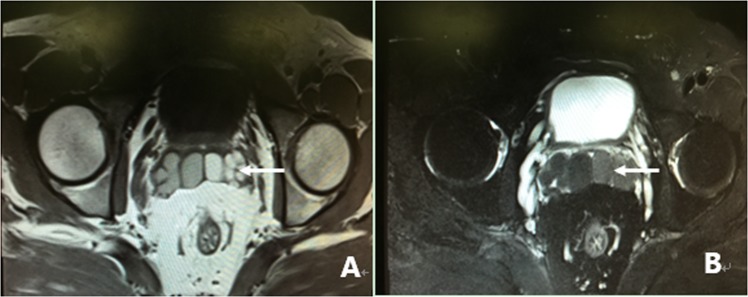
MRI image of EDO. (**A**) The image shows obvious dilatation or enlargement in both sides of the SV. The SV presents intermediate-high signal intensity on T1WI. (**B**) Both sides of the SV present as low signal intensity on T2WI. This pattern suggests that EDO is accompanied with a fresh hemorrhage in both sides of the SV.

All 305 patients underwent examination by MRI. Of the 305 patients, 86.2% (263/305) of the patients had characteristic signs, such as abnormal signal intensity of the seminal vesicle (SV), cystic lesions in the ED area or expansion or enlargement of the SV. The positive findings including the characteristic changes and their frequencies are summarized in Table 2. These changes can be divided into the following four groups: (1) The most common sign viewed by MRI was abnormal signal intensity in the SV area, and it was observed in 62.0% (189/305) of patients, in which 26.2% (80/305) of the patients presented medium to high signal intensity on T1-weighted images and low signal intensity on T2-weighted images in unilateral or bilateral SV. This is opposite to the normal signal intensity of SV imaging, and those patients with unusual signal intensities were confirmed to have had fresh hemorrhages in the SV by seminal vesiculoscopy (Fig. 1). The other 35.7% (109/305) of the patients had middle to high signal intensity on both T1-weighted and T2-weighted images, and those were confirmed to have old hemorrhages in the SV by seminal vesiculoscopy (Fig. 2). (2) The second most common change was cystic lesions in the ED area, which were present in 36.7% (112/305) patients, with or without differences in internal signal intensity or dilatation of the SV. The cystic lesions are divided into the following four categories. Of the 36.7% (112/305) patients, 31.5% (96/305) of the patients had cysts located in the medial region of the verumontanum, of which 25.6% (78/305) of the cases were prostatic utricular cysts (PUC) with sizes ranging from 0.6 × 0.7 cm to 2.0 × 2.3 cm, which was mostly confined to the prostate gland (Fig. 3), and 5.9% (18/305) of the cases were Müllerian duct cysts (MDC) with sizes ranging from 3.4 × 4.5 cm to 8.8 × 11.5 cm, which was usually larger than PUC and beyond the range of the posterosuperior margin of the prostate (Fig. 4), respectively, all of which were confirmed by seminal vesiculoscopy. ED cysts (EDC), a rare type of cyst, were observed in only 1.6% (5/305) of the patients (3 on the left side, 2 on the right side), which communicates with the urethra and one side of the SV. SV cysts (SVC), which the normal typical honeycomb-like and small tubular structure within SV was replaced by single cavity of enlarged cystic structures with smooth edges and clear boundaries, were observed in 3.6% (11/305) of the patients (Fig. 5). The signal intensity of cysts may be normal or abnormal. Among the 36.7% (112/305) cases of cysts in the ED area, 20.3% (62/305) were accompanied by hemorrhages in the cysts or SV, of which 15 were fresh and 47 were old. (3) The third common feature was the changes in size and morphology in the unilateral or bilateral SV, which presented in 32.1% (98/305) of the patients (Fig. 6). The width of the SV more than 1.7 cm or diameter of the inner tubular structure larger than 5 mm was presumed to be indicative of enlargement or dilatation according to our previous study^5^. These differences in morphology or size in the 98 patients were present in unilateral or bilateral SV, with or without internal signal intensity changes, and the average width of the SV was 2.15 ± 0.36 cm. The changes were confirmed to be due to incomplete or complete EDO on the corresponding side by transurethral observation. (4) The fourth sign was stone formation in SV or in an adjacent cyst, which was present in 8.5% (26/305) of the patients. Such stones are most common in SV or prostatic utricle (PU). Some patients showed more than one abnormal sign. There were 13.8% (42/305) of patients whose pelvic MRI images presented no significant abnormalities in the SV area relative to the normal pelvic MRI images.

**Table 2:** MRI image characteristics of 305 patients with refractory hematospermia and/or EDO.

Performance of MRI image	No. of patients	Percentage
Signal intensity in SV	189	62.0
With new hemorrhage	80	26.2
With old hemorrhage	109	35.7
Cysts in the ED area	112	36.7
Prostatic utricle cyst (PUC)	78	25.6
Müllerian duct cyst (MDC)	18	5.9
Seminal vesicle cyst (SVC)	11	3.6
Ejaculatory duct cyst (EDC)	5	1.6
Dilatation or enlargement of SV (width ≥1.7 cm or the inner tubular duct of SV >5 mm)	98	32.1
Stone in the SV or in an adjacent cyst	26	8.5
Cysts accompanied by SV or cyst hemorrhage	62	20.3
With new hemorrhage	15	4.9
With old hemorrhage	47	15.4
Normal	42	13.8

The transurethral endoscopic observation and any related procedures performed by seminal vesiculoscopy were executed successfully in 296 patients, and 9 patients were unable to be treated because of atrophy or abnormal anatomy of the ED area, and all four of the modes of approach for seminal vesiculoscopic insertion have been tried, but no ED or SV was found. The operating time was 20 to 70 min (mean time, 31.6 ± 11.9 min), blood losses were 0 to 10 ml (mean blood loss, 6.3 ± 2.1 ml). The following procedures were performed: (1) Fenestration in PU + seminal vesiculoscopy was performed in 66.6% (197/296) of cases. A pair of symmetrical translucent membranous weak areas was usually observed at the rear 4 and 8 o’clock positions of PUC under endoscopic observation, which is the anatomical basis for the PU fenestration. (2) Seminal vesiculoscopy through the pathological opening in PU was performed in 10.8% (32/296) of cases; unilateral or bilateral ED ectopic openings in the PU were found in 32 patients in this study. Their MRI signal intensities in PU were similar to that of the affected side of SV; (3) TURED + seminal vesiculoscopy were performed in 12.8% (38/296) of cases; (4) seminal vesiculoscopy through the natural orifices in the ED were performed in 2.4% (7/296) of cases; and (5) Electroexcision and fulguration to the abnormal blood vessels or cavernous hemangioma at the posterior urethra, which is usually located at 6 o’clock in the lithotomy position and between the distal end of the verumontanum and the external urethral sphincter, were performed in 7.4% (22/296) of cases. All 18 of the patients with MDC were treated by extensive fulguration or laser cauterization to the cyst inner wall. Intraoperative tissue biopsies of the suspicious lesions in the SV area were performed, and the pathological results were confirmed as chronic nonspecific inflammation without any tuberculosis or tumors in the mucous membranes of the SV. The cavernous hemangioma or abnormal blood vessels (varicosities) at the posterior urethra in these 22 patients were also confirmed by pathological investigation. In addition, single or multiple yellow-brown stones or calcification were found in the SV or the cysts in 8.5% (26/296) of cases, with sizes ranging from 0.2 × 0.3 cm to 0.5 × 1.0 cm. All the stones were successfully removed using grasping forceps or baskets or flushed out after lithotripsy using a holmium laser.

In the 42 refractory hematospermia patients with no positive radiographic findings, this study showed that most of the patients still had obviously abnormal changes in structure or function as seen on endoscopic observation. Cavernous hemangioma or vascular anomalies at the posterior urethra were found in 22 cases who accompanied with hematuria after sexual stimulation, and the other 20 cases showed a certain degree of incomplete unilateral or bilateral EDO, which were confirmed by seminal vesicle massage and management by fenestration in PU.

Of the 296 patients who underwent successful transurethral endoscopic treatment or related procedures, 271 patients were successfully followed up for 6 to 72 months (mean, 32.4 ± 23.6 months). The other 25 patients were lost to follow-up. The hematospermia of all 271 patients disappeared within 2 to 6 weeks, and 93.0% (252/271) of the patients did not experience further hematospermia during the follow-up period. The symptoms of the patients who experienced ejaculatory pain, discomfort, or pain in the lumbosacral or perineal region and other symptoms were also significantly alleviated or disappeared. Here, 7.0% (19/271) of the patients experienced repeated recurrence of hematospermia after 5–20 months, and 9 of them accepted a second round of treatment by seminal vesiculoscopy and recovered within 1–2 months of the second operation and remained symptom-free during the more than 12 months of follow-up. The rest of them preferred oral medication, and the hematospermia symptom still recurred repeatedly. In terms of complications, 4.8% (13/271) of the patients over 40 years complained of a slight decrease in their orgasmic intensity after the operation. Here, 5.9% (16/271) of patients said that the volume of each ejaculation was greater, but its ejaculate consistency was thinner after the operation. All of these cases of differing ejaculate were due to previous treatment by TURED/TUIED. Two patients with mild preoperative urethral stricture complained that they had gotten worse after their operations. Only one patient experienced slight unilateral epididymitis after the operation and recovered quickly after being treated by antibiotics. No other obvious postoperative complications such as perineal pain, rectal injury, retrograde ejaculation, urinary incontinence, urinary tract infection, or clinically significant hematuria was observed in any of the four surgical vesiculoscopy procedures. The quality of semen was evaluated in only the 19 cases accompanied by infertility and it was improved significantly after the operation. CASA analysis at 1 to 6 months after operation showed that the volume of semen increased from 0–1.5 ml (0.87 ± 0.31 ml) to 1.5–4.5 ml (2.25 ± 0.80 ml), the sperm densities were increased from 0–15.3 × 10^6^/ml (4.44 ± 4.63 × 10^6^/ml) to 21.5–63.0 × 10^6^/ml (35.21 ± 11.71 × 10^6^/ml), and the percentage of grades A + B spermatozoa were increased from 0–33.4% (8.13 ± 9.82%) to 27.4–66.7% (43.75 ± 10.60%), all of which were significantly increased compared with before the operation (p < 0.001) (Table 3).

**Table 3:** Semen analysis results of 19 patients with refractory hematospermia and infertility before and after operation.

	Before surgery	1–6 months after surgery	p value
Volume (mL)	0.87 ± 0.31	2.25 ± 0.80	0.001
Concentration (×10^6^/ml)	4.44 ± 4.63	35.21 ± 11.71	0.001
Grades A + B (%)	8.13 ± 9.82	43.75 ± 10.60	0.001

## Discussion

Hematospermia usually presents as a benign, self-limiting course, which can be managed only by simple reassurance, basic investigation or other conservative methods, including anti-inflammation, anti-infection, and hemostatic therapy^8–10^. However, a small number of patients with consistent or intermittent hematospermia refractory to a variety of conservative treatments have experienced great anxiety and psychological concern. Previous studies have shown that the etiology of hematospermia is varied and complicated, including inflammation or infection of the urogenital tract, lithiasis, cysts, obstruction, neoplasms, vascular anomalies of the posterior urethra, trauma, and iatrogenic and systemic origins^4^. Some relatively common factors include inflammation, infection, and EDO caused by cysts or stones^4,11–14^. Inflammation and infection are more commonly observed in patients younger than 40 years of age and accounted for 39% to 55% of all hematospermia^8^. The recurrent and refractory hematospermia which appear as a protracted course of disease are closely related to the characteristics of the local anatomy and physiology. Only an accurate understanding of the pathological features of the ED area of the patients with refractory hematospermia can render the etiology clear and the corresponding treatment more effective.

Three-dimensional imaging of MRI has excellent resolution and can distinguish soft tissue structure and show the hemorrhagic peculiarity in SV and the prostate area and the specific morphological characteristics, which gives it considerable value for the guidance of etiological diagnosis and treatment planning for refractory hematospermia. MRI is considered as the gold standard for the hematospermia imaging examination^5,15,16^. As far as we know, the current work has the largest sample size of any report of systematic MR imaging studies on hematospermia patients. According to the MRI results, the characteristic imaging changes mainly presented as the following four types: Abnormal signal intensity of the SV area, morphology and size changes of the unilateral or bilateral SV (width ≥1.7 cm, diameter of the inner tubular structure >5 mm), cystic lesion in the adjacent ED area, and stone formation in the SV or in an adjacent cyst. Patients may have one or more types of characteristic changes in the SV area. The common cystic lesions in the ED area are PUC, MDC, EDC, and SVC. The signal intensity of cysts may be normal or abnormal. The PUC is the most common cyst among the aforementioned cysts and presents in 25.6% (78/305) of the patients, followed by MDC which presents in 5.9% (18/305) of the patients. EDC and SVC are relatively rare in this group of patients. Eight cases of refractory hematospermia accompanied by azoospermia in this study were found to be complete EDO, which were confirmed by observing no expressed SV fluid upon transrectal SV massage. Twenty two cases of refractory hematospermia accompanied by obvious hematuria after ejaculation were observed as having posterior cavernous urethral hemangioma or abnormal blood vessels which were located at about 0.5 to 1 cm away from the distal end of the verumontanum or the plane of external urethral sphincter. All of these cases were confirmed by the characteristics of no abnormal finding on MRI, no expressed SV fluid or bleeding upon transrectal SV massage, appearance of hemangioma-like changes at the posterior urethral area, and disappearance of symptoms after operation. Then, all of the other hematospermia patients were defined as incomplete EDO, which were confirmed by several lines of evidence: cystic dilatation of the SV, formation of cysts or stones in the seminal duct area, formation of pathological openings of the ED in PU, and/or difficulty to detect expressed SV fluid during transrectal SV massage. Although it is difficult to define exactly the existence of incomplete EDO based on present clinical techniques because of a lack of unified diagnostic criteria and recognized diagnostic methods, this study strongly suggested that incomplete EDO due to different reasons is a very universal characteristic in refractory hematospermia patients.

The obstruction, inflammation, and stones can exacerbate each other and constitute the pathological basis of the refractory hematospermia. For such cases, conservative treatment is often ineffective and surgical intervention to relief obstruction and stone is usually required. In this way, the predominant factors associated with refractory hematospermia should be the EDO caused by the cysts or stones in the ED area^17,18^. This conclusion is consistent with a previous study by Zhang *et al*.^19^. Although refractory hematospermia in patients aged >40 years may be caused by the tumors of the reproductive system, a literature review about the etiology of hematospermia showed that 3.5% (33/931) of the patients to be attributable to the tumors^9^. Prior to this study, we found only three cases of prostate cancer in the primary screening of patients with refractory hematospermia. No any other tumor was found in the finally included 305 patients.

Some studies into safe and effective use of seminal vesiculoscope techniques have confirmed their advantages, specifically minimal invasion, few postoperative complications, and significant effectiveness^20–22^. Yang *et al*.^23^ first reported the use of seminal vesiculoscopy in patients with refractory hematospermia for the diagnosis and treatment and confirmed this technique to be safe and feasible. Han *et al*.^24^ reported the diagnosis and treatment of seminal vesiculoscopy in 70 patients with refractory hematospermia. Over the average 1-year follow-up, 78.6% of patients with refractory hematospermia showed improvement, with a recurrence rate of 10%. Liu *et al*.^7^ used F7 rigid ureteroscopes on the management of 114 cases of refractory hematospermia and showed the cure rate to be 94.4%. Chen *et al*.^6^ recently reported the largest series of transurethral seminal vesiculoscopy cases, and demonstrated satisfactory short-term symptomatic improvements with an 85.0% alleviation rate or rate of disappearance of hematospermia in 324 patients within 3 months after surgery. We here verified that this technique is safe and has a lower complication rate. It has significant long-term efficacy, and the recovery rate of refractory hematospermia was 93.0% after the first round of treatment. This recovery rate was found to be improved upon a second round of treatment. At the same time, this study showed the technique to be associated with better-quality semen and a higher rate of natural pregnancy in couples with EDO-induced infertility. It also should be mentioned that all the patients in this group had been treated by many conservative methods for more than one month. These methods included different antibiotics, so although it has been reported that the most common reason for hematospermia is infection or inflammation, but we did not find any obvious infections in any of the patients’ seminal tracts in the operation, nor did we find significant postoperative complications related to infection. This observation could be related to previous antibiotic treatment.

Transurethral seminal vesiculoscopy and related procedures are minimally invasive operations with some degree of difficulty and risk^7^. Although there have been some reports on the clinical applications of seminal vesiculoscopy in recent years, there are still some important issues that should be discussed. Compared with other studies, our study has a series of characteristics and advantages in the etiological analysis and treatment of hematospermia, and we provide much new information, as follows: (1) The combined applications of MRI features and endoscopy technology can identify most of the common causes of refractory hematospermia, even for patients without obvious MRI changes. (2) We are the first group to propose that transrectal SV massage is a reliable and simple technology for the identifying the ED orifice in seminal vesiculoscopy. This simple procedure can provide valuable information for identifying the cause of hematospermia, locating the lesion, and selecting the mode of seminal vesiculoscopy. Considerable milky white, jelly-like liquid can be expressed from both ED orifices under normal situations. However, SV fluid can be difficult to express or can even be undetectable in incomplete or complete EDO cases. (3) Although there is author who advocates that seminal vesiculoscopy via the natural pathway should be the first selected approach^6^, we propose, based on the very fine nature of the ED and its ease of restenosis and repeated obstruction, that an approach through the PU should still be the first choice for most hematospermia patients. (4) Cysts in ED areas should be dealt with by incision, excision, or fulguration to the inner wall of the cyst according to the type of cyst. In dealing with MDC, the patency of the ED and SV should also be controlled and confirmed by SV massage to remove any abnormal conditions. For lesions that may be malignant, biopsies should be performed under endoscopy. (5) For the prevention of postoperative complications, we suggest that early SV massage and early recovery of ejaculation once or twice a day are of great value in reducing inflammatory adhesions in the ED area and avoiding the recurrence of hematospermia.

In summary, MRI examination and subsequent endoscopic observation of this group of refractory hematospermia confirmed that the primary causes are the complete or incomplete EDO caused by inflammation or infection, different kinds of cystic lesion, and formation of stones within the ED area. MRI examination can accurately display the abnormal signal characteristics, structural change in the ED region and provide important guidance for diagnosis and further treatment. As a recent emerging technique, it has shown a significant effect on the diagnosis and treatment of refractory hematospermia and EDO attributable to different, causes including stones in the seminal duct and the cysts in the ED area. It has few complications and significant long-term efficacy, which gives it wide prospects for application in the management of common diseases in the distal region of the seminal tract. At the same time, the limitations of this technique should also be considered. For instance, a few patients may not be able to undergo endoscopic examination and treatment successfully. In addition, minor damage to the distal region of the seminal tract may lead to the re-stenosis and EDO, resulting in the recurrence of hematospermia. Therefore, the surgeons should be proficient in endoscopic operations and very cautious during their preliminary attempts with this technique.

## Methods

### Clinical Data

In this study, all the patients with persistent or recurrent hematospermia or hematospermia accompanied with infertility lasting more than 6 months and without any visible responses to conservative treatment, including long-term anti-infection, anti-inflammatory, and hemostatic therapy who visited our hospital from Jan 2009 to Feb 2017, were investigated by a series of routine examinations, including blood tests, urinalysis, coagulation function, hepatic and renal function, digital rectal examinations (DRE), semen analysis (CASA), biochemical testing of seminal plasma, and imaging examinations including TRUS and MRI. Informed consent for every patient was obtained. The serum prostate specific antigen (PSA) was also measured in men over 40 years of age, and transrectal ultrasound-guided prostate biopsy was performed in the patients with abnormally high PSA for further evaluation so as to exclude some serious pathological conditions. In the end, three cases of prostate cancer and five cases of abnormal liver function or patients on oral anticoagulant therapy were excluded from this study, and 305 patients with benign persistent or recurrent hematospermia were enrolled in this study. Nineteen patients who were referred to the hospital with refractory hematospermia accompanied by infertility were also included in this study.

### Surgical Procedure

**Figure 7:**
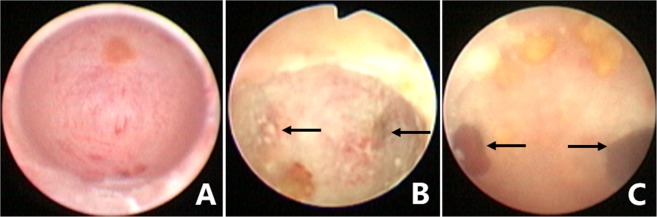
Different patterns under endoscopic observation in the PU. (**A**) The normal oval inner morphology of PU in some patients. (**B**) A pair of symmetrical translucent membranous weak areas was found at the 4 and 8 o’clock positions on the posterolateral wall of the PU in some patients. A small stone was also observed in the PU. (**C**) Bilateral ectopic passages of the EDs were observed at the 4 and 8 o’clock positions of the posterolateral wall of the PU in pathological conditions. Some small stones were observed in the PU.

**Figure 8:**
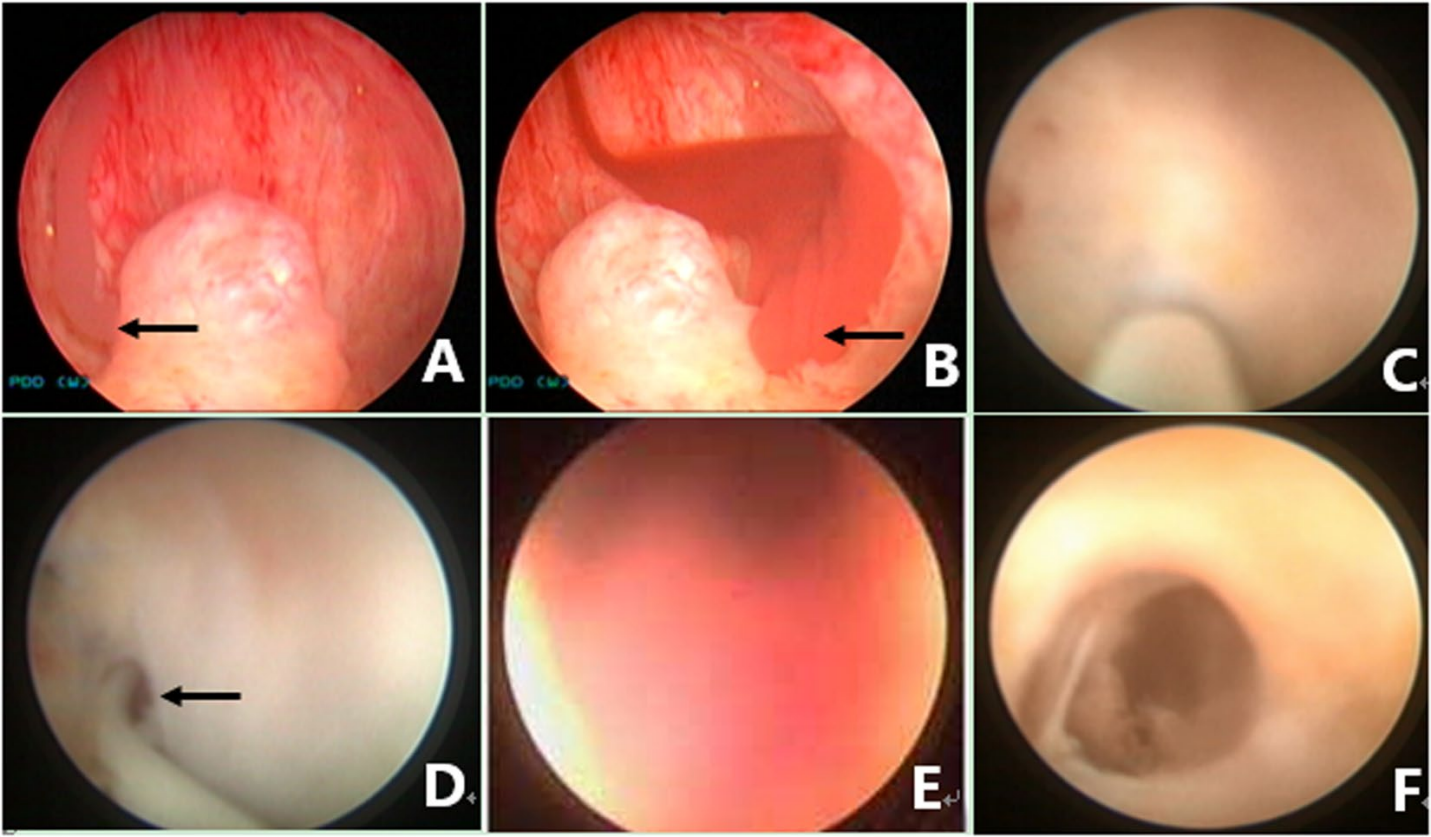
Seminal vesiculoscopy and treatment by fenestration in the PU. (**A**) Endoscopic observation confirmed that the hemorrhage was not located on the right SV because normal jelly-like seminal fluid poured from the right ED orifice upon seminal vesicle massage. (**B**) Bloody fluid poured from the left ED orifice upon seminal vesicle massage, which indicated that the hemorrhage was located in the left SV. (**C**) A translucent membranous weak area was identified at the 4 o’clock position on the posterolateral wall of the utricle. (**D**) The soft tip of a guidewire was inserted along the channel without resistance for 3–5 cm, indicating that the tip had been inserted smoothly into the left SV. (**E**) The seminal vesiculoscope was inserted into the SV under the guidance of a guidewire. The SV was filled with a large quantity of red seminal fluid. (**F**) After irrigation and observation of the SV, the seminal vesiculoscope was withdrawn from the SV upon the formation of an ED short opening.

**Figure 9:**
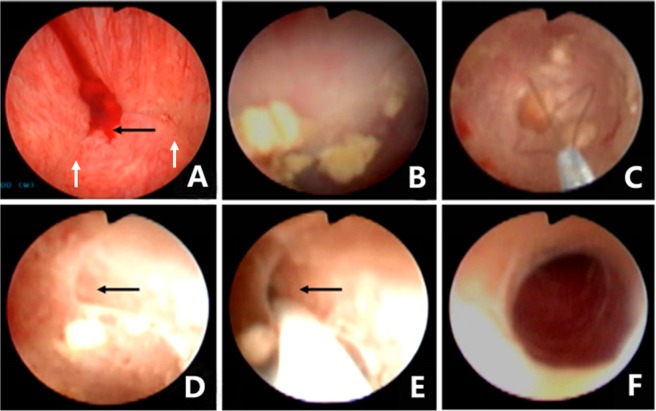
Seminal vesiculoscopy and treatment by the pathological opening in PU. (**A**) Bloody fluid poured from the orifice of the PU upon bilateral seminal vesicle massage, but nothing came from either of the ED orifices (white arrow). (**B**) The seminal vesiculoscope was inserted into the PU, and several stones were found in the PU. (**C**) The stones were removed by grasping forceps. (**D**) There are pathological openings in the PU at the 4 and 8 o’clock area in the PU (showing only the 4 o’clock opening). (**E**) The guidewire can be directly inserted into the SV through the opening. (**F**) The seminal vesiculoscope was inserted into the SV.

**Figure 10:**
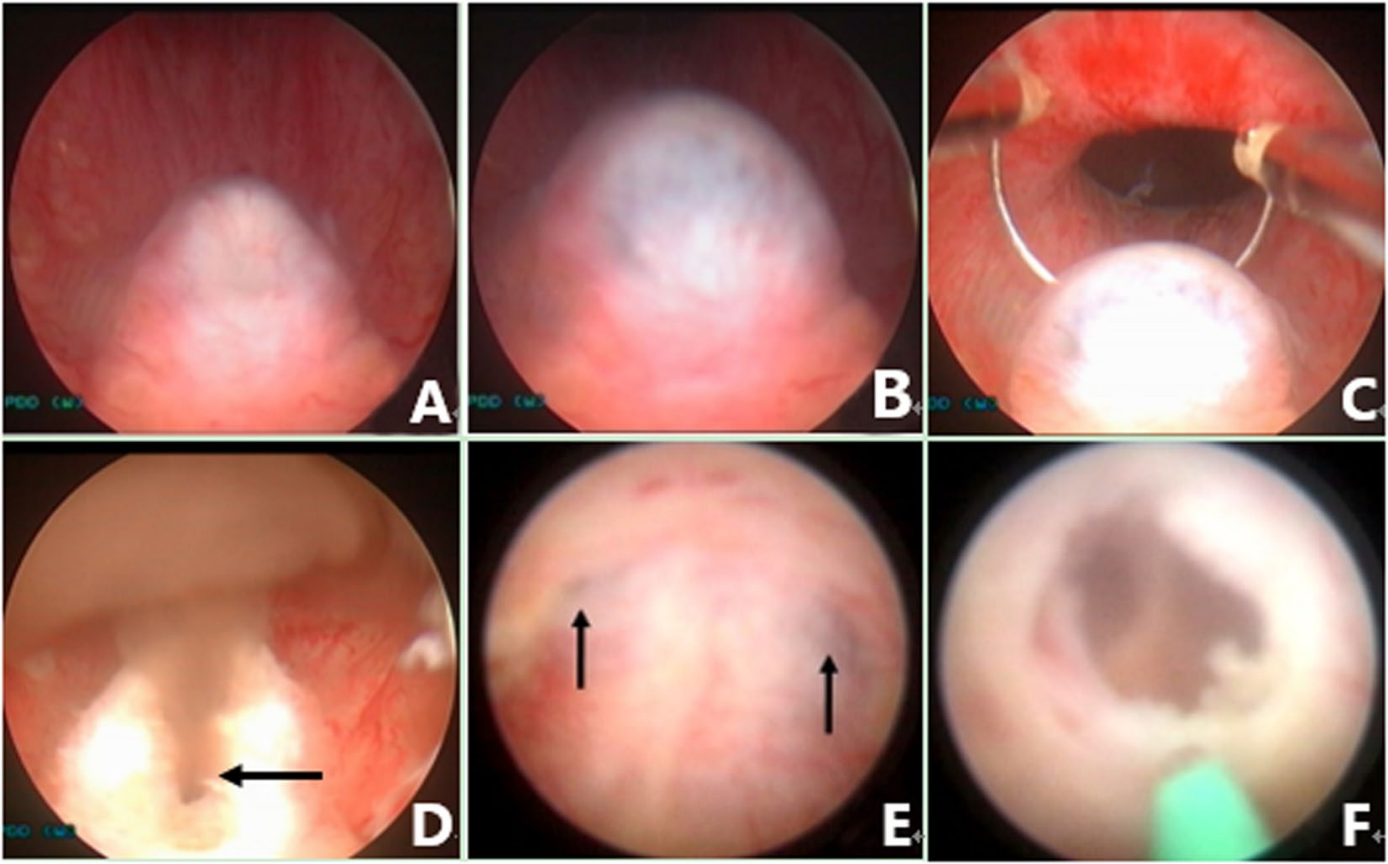
Seminal vesiculoscopy and treatment based on TURED. (**A**) Nothing came from either the ED orifice or the orifice of the PU upon bilateral SV massage, indicating the patients had complete EDO. (**B**) Transrectal massage to the medial area of the prostate showed that the PU opening is obstructed, and the verumontanum presented as a cystic mass with fluctuation. (**C**) Transurethral deroofing resection of the verumontanum was performed. (**D**) After transurethral deroofed resection of the verumontanum, chocolate-like fluid was observed emerging from the orifice of the PU. (**E**) Translucent membranous weak areas were identified at the 4 and 8 o’clock positions on the posterolateral wall of the utricle. (**F**) A holmium laser was used for fenestration.

This study was approved by the Daping Hospital Ethics Committee, and all study procedures met the stipulations of the WMA Declaration of Helsinki. All patients underwent the seminal vesiculoscopy and related procedures using an F25.6 electroresectoscope (Gyrus ACMI, Southborough, MA, USA, or Olympus, Tokyo, Japan) and an F4.5–7.5 ureteroscope (Wolf, Germany, or Olympus, Tokyo, Japan) under epidural or lumbar anesthesia in the lithotomy position. First, the resectoscope was slowly and gently threaded through the urethra and into the bladder. The orifices of the ureters and bladder mucosa were observed to exclude any suspicious abnormality of the bladder. Then the resectoscope was withdrawn to the posterior urethra and verumontanum area. The morphology of verumontanum, the orifices of the PU and ED, bleeding, and vascular abnormalities and neoplasms in the prostatic urethra were observed. The orifice of ED in most of the patients could be identified by the expression of SV fluid during the transrectal SV massage. However, expressed SV fluid could be difficult to detect or could even be undetectable in the incomplete or complete EDO cases. The orifices of the ED are usually located on both sides of the verumontanum hillock, 2 mm away from the orifice of the PU, showing either a triangular or linear arrangement with the orifice of the PU. The following four approach modes were usually used for the seminal vesiculoscopic insertion into the SV: 1. Fenestration approach by PU: The F4.5–7.5 ureteroscope can be guided under the zebra guidewire or inserted directly into the PU. Continuous sodium chloride (0.9%) irrigation, using a peristaltic pump, was usually used to obtain and sustain a clear operative visual field. The irrigation pressure and flow rate were set at 200 mmHg and 0.1–0.2 L/minute, respectively. The flow rate could be decreased to avoid excessive intra-seminal vesicle irrigation by adjusting the rotary knob on the ureteroscope. A pair of symmetrical translucent membranous weak areas was usually found at the 4 and 8 o’clock positions on the posterolateral wall of the utricle, which is the closest point between the walls of the ED and PU (Fig. 7). A zebra guidewire or ureteral catheter was used to puncture these weak areas. If the tip of the zebra guidewire or ureteral catheter broke through the weak area easily and could be pushed to a depth of 3–5 cm, the tip was considered to have been inserted into the SV, and then the vesiculoscope was inserted into the SV under the guidance of the zebra guidewire or the ureteral catheter (Fig. 8). Sometimes a holmium laser could also be used for fenestration in PU. 2. The pathological opening was approached through the PU (Fig. 9): Bilateral or unilateral ectopic passages of the EDs could be found at the 4 and 8 o’clock positions of the posterolateral wall of the utricle in some patients because of previous pathological factors (Fig. 7). The vesiculoscope can be directly inserted into SV through the pathological opening for the observation and treatment. 3. Natural approach through the orifices of ED: The orifices of ED are not completely obstructed in most of the hematospermia patients, which usually can be identified by SV massage. The zebra guidewire or ureteral catheter could be inserted directly into the orifice in some patients. When this was the case, an F 4.5 vesiculoscope could then be inserted into the SV along with the guidewire, and then an F 6.0 vesiculoscope was usually inserted into the SV for the ED dilation. 4. The natural approach was used after TURED or TUIED (Fig. 10). In some pathologic situations, the orifice of the ED and PU was completely obstructed or had disappeared because of local inflammation, infection, or damage from the adjacent cyst. It is difficult to insert the zebra guidewire or the ureteral catheter into both the orifices of ED or the PU, while the resectoscope can be used carefully to perform a slight resection or incision of the verumontanum and the orifices of the ED, at a depth of about 3–5 mm. After removal of the verumontanum, the orifice of the ED can be identified directly or combined with the SV massage. Then a zebra guidewire may be inserted into the orifice of ED and the vesiculoscope can be inserted successfully.

**Figure 11:**
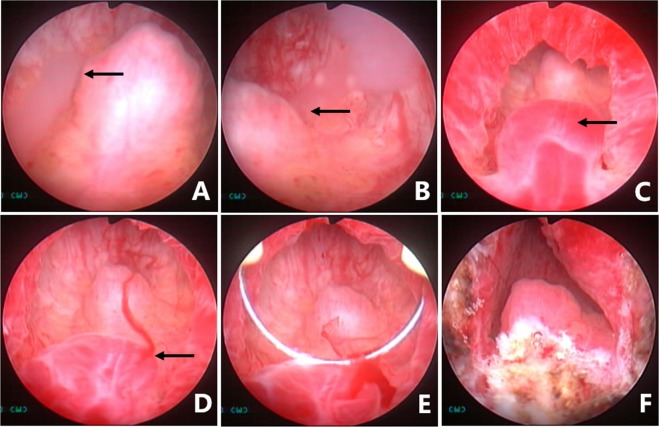
Management of a cavernous hemangioma at the posterior urethra. (**A**) Normal white jelly-like seminal fluid poured from the right ED orifice upon seminal vesicle massage. (**B**) Normal white jelly-like seminal fluid poured from the left ED orifice upon seminal vesicle massage. (**C**) An obvious cavernous hemangioma with a size of about 5 × 5 mm was seen at about 5 mm away from the distal margin of the verumontanum. (**D**) Active bleeding was observed on the cavernous hemangioma. (**E**) The cavernous hemangioma was resected and/or fulgurated with an electric cutting loop. (**F**) The verumontanum area after the hemangioma was resected and/or fulgurated.

During the endoscopic operation, it is usually necessary to irrigate with a pressure pump to maintain a clear view, but the perfusion pressure should be controlled, to avoid reflux infections such as epididymitis. Different procedures can be performed to treat the diseases in the ED and adjacent areas, depending on the pathological changes in different cases. Cysts in the ED area, including PUC, MDC, and SVC, can be treated by incision, fulguration, or extensive cautery to the cyst wall, for large cysts. Small stones in the ED, PU, or SV in some patients should be removed with grasping forceps or baskets, or should be flushed out after lithotripsy by holmium laser. Hematospermia in the SV or PU was irrigated and potentially abnormal tissue was biopsied. The abnormal changes in blood vessels or cavernous hemangiomas in the urethra of the prostate were resected, fulgurated, or both (Fig. 11). After the procedure, a urethral Foley catheter was placed overnight. Daily SV massage was performed, usually by the doctor or by the patient’s wife or relative for the first 10–14 days after the operation. Early regular ejaculation was usually encouraged to reduce the chance of inflammatory adhesions or re-obstruction. Perioperative antibiotics were usually prophylactic used for about 2–3 days.

### Statistical Analysis

The duration of the operation, blood loss, parameters of semen analysis, and width of the SV were calculated and are presented as average ± standard deviation. The prevalence of the different morphological features of the bilateral SV and ED regions in MRI, different approach modes for the seminal vesiculoscopic insertion, the effects of seminal vesiculoscopy for the treatment of hematospermia, recurrence rate, postoperative complications, and the pregnancy rates (excluding those with artificial assisted fertilization) were recorded and are presented as percentages. The semen parameters before and after the operation were compared by Student’s t-test or χ^2^-test. p < 0.05 was considered statistically significant. The statistical analysis was performed with the SPSS 16.0 software package (SPSS, Chicago, IL, USA).
